# Author Correction: Synthesis of vitamin D3 loaded ethosomes gel to cure chronic immune-mediated inflammatory skin disease: physical characterization, in vitro and ex vivo studies

**DOI:** 10.1038/s41598-024-77479-3

**Published:** 2024-10-29

**Authors:** Yasir Mehmood, Hira Shahid, Shabbir Ahmed, Anjum Khursheed, Talha Jamshaid, Muhammad Jamshaid, Atrsaw Asrat Mengistie, Turki M. Dawoud, Farhan Siddique

**Affiliations:** 1 Department of Pharmaceutics, Faculty of Pharmaceutical Sciences, Government College University, PO.Box 38000, Faisalabad, Pakistan; 2 Department of Pharmacology, Faculty of Pharmaceutical Sciences, GC University, PO.Box 38000, Faisalabad, Pakistan; 3 Riphah Institute of Pharmaceutical Sciences (RIPS), Riphah International University, PO.Box 38000, Faisalabad, Pakistan; 4 Fatima College of Health Sciences, PO.Box 36330, Toba Teksingh, Punjab Pakistan; 5 Faculty of pharmacy, Grand Asian University, Pasrur road, PO.Box 51410, Sialkot, Punjab Pakistan; 6 Department of Pharmaceutics, Faculty of Pharmacy, The Islamia University of Bahawalpur, Bahawalpur, Pakistan; 7 Faculty of Pharmaceutical Sciences, UCP, PO.Box 51410, Lahore, Punjab Pakistan; 8 Department of Biology, Bahir Dar University, P.O.Box 79, Bahir Dar, Ethiopia; 9 Department of Botany and Microbiology, College of Science, King Saud University, P. O. BOX 2455, 11451 Riyadh, Saudi Arabia; 10 School of Pharmaceutical Science and Technology, Tianjin University, Tianjin, P.R. China

Correction to: *Scientific Reports *10.1038/s41598-024-72951-6, published online 12 October 2024

In the original version of this Article Atrsaw Asrat Mengistie was incorrectly affiliated with ‘Department of Botany and Microbiology, College of Science, King Saud University, P. O. BOX 2455, 11451 Riyadh, Saudi Arabia.’ Their correct affiliation is listed below.

Department of Biology, Bahir Dar University, P.O.Box 79, Bahir Dar, Ethiopia.

The original Article has been corrected.

